# Representation of the QM Subsystem for Long-Range Electrostatic Interaction in Non-Periodic Ab Initio QM/MM Calculations

**DOI:** 10.3390/molecules23102500

**Published:** 2018-09-29

**Authors:** Xiaoliang Pan, Edina Rosta, Yihan Shao

**Affiliations:** 1 Department of Chemistry and Biochemistry, University of Oklahoma, 101 Stephenson Parkway, Norman, OK 73019–5251, USA; panxl@ou.edu; 2 Department of Chemistry, King’s College London, London SE1 1DB, UK; edina.rosta@kcl.ac.uk

**Keywords:** electrostatics, multipolar expansion, multiscale modeling, QM/MM

## Abstract

In QM/MM calculations, it is essential to handle electrostatic interactions between the QM and MM subsystems accurately and efficiently. To achieve maximal efficiency, it is convenient to adopt a hybrid scheme, where the QM electron density is used explicitly in the evaluation of short-range QM/MM electrostatic interactions, while a multipolar representation for the QM electron density is employed to account for the long-range QM/MM electrostatic interactions. In order to avoid energy discontinuity at the cutoffs, which separate the short- and long-range QM/MM electrostatic interactions, a switching function should be utilized to ensure a smooth potential energy surface. In this study, we benchmarked the accuracy of such hybrid embedding schemes for QM/MM electrostatic interactions using different multipolar representations, switching functions and cutoff distances. For test systems (neutral and anionic oxyluciferin in MM (aqueous and enzyme) environments), the best accuracy was acquired with a combination of QM electrostatic potential (ESP) charges and dipoles and two switching functions (long-range electrostatic corrections (LREC) and Switch) in the treatment of long-range QM/MM electrostatics. It allowed us to apply a 10Å distance cutoff and still obtain QM/MM electrostatics/polarization energies within 0.1 kcal/mol and time-dependent density functional theory (TDDFT)/MM vertical excitation energies within 10^−3^ eV from theoretical reference values.

## 1. Introduction

It has long been recognized that a rigorous treatment of long-range electrostatic interactions is essential for modeling biological processes in the condensed phase [1,2,3]. In classical simulations of biological systems, the Ewald summation family of methods, the particle mesh Ewald (PME) method especially [4,5], has become the de facto standard way to handle the long-range electrostatics.

The PME method was originally developed for use with molecular mechanics (MM) force field calculations [6], but long-range electrostatics is equally important [7] in combined quantum mechanical molecular mechanical (QM/MM) simulations; in such simulations [8,9,10,11], a key region of the system is handled quantum mechanically, while an MM description for the rest of the system. As far as the treatment of QM/MM electrostatics is concerned, QM/MM simulations fall into two categories. In the “mechanical-embedding” (ME) QM/MM simulations, the electrostatic interactions between the QM and MM subsystems retain a description at the MM level. This is theoretically unappealing and practically error-prone, because it totally ignores the perturbation of the QM wavefunction by the MM atoms (no matter how close they are). As a result, there has been a decline in the use of the mechanical-embedding QM/MM calculations.

In the “electrostatic-embedding” (EE) QM/MM simulations, on the other hand, the QM electron density interacts explicitly with the MM charges (or multipoles). In most EE-QM/MM calculations, this is conveniently implemented by augmenting the one-electron core Hamiltonian of the QM region with the electrostatic potential of MM charges (or multipoles). In QM calculations using Gaussian basis sets, for example, this necessitates the evaluation of additional three-center integrals, each of which is a Coulomb integral between two Gaussian basis functions and one MM charge/multipole.

In EE-QM/MM simulations, a multipolar representation is sometimes adopted for the QM electron density. This happens mostly in the context of QM/MM calculations under the periodic boundary conditions (PBC). For example, within the QM/MM Ewald [12] and PME methods [13], Mulliken charges were employed to represent the semi-empirical QM electron density in the reciprocal-space (i.e., long-range electrostatics) calculation. A Mulliken charge representation was also adopted in semi-empirical QM/MM simulations on extended systems using the isotropic periodic sum [14] and Wolf summation [15] methods. In ab initio Gaussian-basis QM/MM PBC calculations, however, the use of Mulliken charges in the reciprocal space was found to cause substantial instability in the calculated SCF energies with medium or large-sized basis sets. Subsequently, the use of CHELPG charges in the reciprocal space was shown to yield more stable ab initio QM/MM Ewald calculations [16,17]. CHELPG and other electrostatic potential (ESP) charges were also used in other implementations of ab initio QM/MM (ai-QM/MM) PBC calculations [18,19,20]. We note that, however, ab initio Gaussian-basis QM/MM PBC calculations have yet to gain wide use.

In non-periodic ab initio QM/MM calculations, whose use remains mainstream, a multipolar representation for the QM electron density is rarely employed for handling the QM/MM electrostatics. One notable exception occurs with ai-QM/MM calculations using the MOLCAS software [21,22], where the electrostatic potential fitted (ESPF) method from Ferré and Ángyán [23] interacts ESP charges and dipoles (from QM atoms) with MM atoms. While it can produce noticeably different results (as is evident from several examples in this article), this alternative approach to QM/MM electrostatic embedding is very attractive in terms of its computational efficiency. It completely removes the need to compute the aforementioned three-center integrals, which can become cumbersome for systems containing tens of thousands of (if not more) MM atoms. Another notable exception occurs with the TINKTEP interface [24] for QM/MM calculations using the AMOEBA force field. There, the QM subsystem adopts an auxiliary multipolar representation (via the distributed multipole analysis) in its interaction with the polarizable environment.

Besides ESPF and TINKTEP, there are a number of other approaches that can speed up the evaluation of ab initio QM/MM electrostatics in a non-periodic calculation: (a) In the long-range electrostatic corrections (LREC) method from Fang, Duke and Cisneros [25,26], one only has to compute Gaussian integrals between QM basis functions and near-field MM charges with a cutoff distance (ca. 22 Å). In order to reproduce PME results, near-field MM charges were scaled using a smoothing function. (b) In the solvated macromolecule boundary potential (SMBP) method from Benighaus and Thiel [27,28] (which is based on the generalized solvent boundary potential (GSBP) method for MM simulations [29] and semi-empirical QM/MM simulations [30]), MM charges within a cutoff distance still interact directly with the electron density, while those outside the radius are approximated as a set of charges on a sphere slightly beyond the cutoff radius. (c) The PME method from Giese and York [31] for periodic QM/MM electrostatics can be extended to handle QM-MM long-range electrostatics in a non-periodic QM/MM calculation (in a way similar to the Fourier transform Coulomb methods [32,33]). Hereby, the embedding potential from remote MM atoms can be computed in the reciprocal space and then interpolated from the FFT grid to the molecular quadrature grid of the QM region. (d) The fast multipole moment (FMM) method [34] can be used to account for the contributions from “far-field” MM atoms, namely those that do not overlap with QM electron density, at a much reduced cost.

In this work, we propose a simple and efficient approach for handling long-range ab initio QM/MM electrostatics in non-periodic calculations, which combines the merits of the SMBP and ESPF methods. Specifically, we will adopt a hybrid scheme, where the QM electron density is used explicitly in the evaluation of short-range QM/MM electrostatic interactions, while a multipolar representation for the QM electron density is employed to account for the long-range QM/MM electrostatic interactions. In order to avoid energy discontinuity at the cutoffs, which separate the short- and long-range QM/MM electrostatic interactions, a switching function will be utilized to ensure a smooth potential energy surface.

This paper is organized as follows. The basic theory of our hybrid scheme will be introduced in Section 2, while the computational details will be included in Section 3. Results for our test systems, neutral and anionic oxyluciferin molecules in aqueous and macromolecular environments, will be presented in Section 4. Finally, in Section 5, concluding remarks will be made.

## 2. Theory

### 2.1. Mechanical Embedding ai-QM/MM Calculations

Within mechanical embedding ai-QM/MM calculations, the QM electron density (together with the nuclei) is usually represented as a set of fixed-value pre-computed point charges $q_{A}^{\mathrm{ref}}$. If the MM environment consists of only point charges $q_{B}$, the corresponding ai-QM/MM electrostatic energy is:

(1) $$E_{\mathrm{QM}/\mathrm{MM}}^{\mathrm{mechanical}}=\sum_{A}\sum_{B}q_{A}^{\mathrm{ref}}q_{B}^{}\frac{1}{\left|r_{A}-r_{B}\right|}\;,$$

where $r_{A}$ and $r_{B}$ refer to the position of QM and MM atoms, respectively.

### 2.2. Electrostatic Embedding ai-QM/MM Calculations

Based on the representation of the QM subsystem, electrostatic-embedding ai-QM/MM methods can be further divided into two sub-categories. In the first sub-category, which is the dominant choice today, the QM subsystem is described with nuclear charges $Z_{A}$ and a distributed electron density ρ(r), which interact explicitly with the electrostatic embedding potential, ϕ(r), from the MM environment,

(2) $$E_{\mathrm{QM}/\mathrm{MM}}^{\mathrm{elec},\;I}=\sum_{A}Z_{A}\phi \left(r_{A}\right)-\int \rho \left(r\right)\phi \left(r\right)dr.$$

If the environment consists of only point charges $q_{B}$, the MM embedding potential is:

(3) $$\phi \left(r\right)=\sum_{B}\frac{q_{B}}{\left|r-r_{B}\right|}.$$

In the second sub-category, the QM subsystem adopts a multipolar representation. Using $M_{Am}$ to represent the *m*-th generalized multipole moment at atomic site *A*,

(4) $$M_{Am}=\left\{\begin{array}{cc} q_{A}, & m=0\\ \mu_{Ax}, & m=1\\ \mu_{Ay}, & m=2\\ \mu_{Az}, & m=3\\ \;\vdots & \end{array}\right.$$

the alternative definition for the system-environment electrostatic interaction energy can be written as:

(5) $$E_{\mathrm{QM}/\mathrm{MM}}^{\mathrm{elec},\;\mathrm{II}}=\sum_{A}\sum_{m}M_{Am}F_{Am},$$

which is a sum of the product between atomic multipoles and the local Taylor expansion of the MM electrostatic embedding potential,

(6) $$F_{Am}=\left\{\begin{array}{cc} \phi_{A}, & m=0\\ E_{Ax} & m=1\\ E_{Ay}, & m=2\\ E_{Az}, & m=3\\ \;\vdots & \end{array}\right.$$

Note that the zeroth-order Taylor expansion, $F_{A0}=\phi_{A}$, is the value of MM electrostatic potential at atomic site *A*, which appeared above in Equation (2) as $\phi (r_{A})$.

If the environment consists of only point charges $q_{B}$, the local Taylor expansion is:

(7) $$F_{Am}=\sum_{B}q_{B}T_{Am,B},$$

where T is the multipole-charge interaction tensor,

(8) $$T_{Am,B}=\left\{\begin{array}{cc} \frac{1}{\left|r_{A}-r_{B}\right|}\;, & m=0\\ \frac{x_{A}-x_{B}}{{\left|r_{A}-r_{B}\right|}^{3}}\;, & m=1\\ \frac{y_{A}-y_{B}}{{\left|r_{A}-r_{B}\right|}^{3}}\;, & m=2\\ \frac{z_{A}-z_{B}}{{\left|r_{A}-r_{B}\right|}^{3}}\;, & m=3\\ \;\vdots & \end{array}\right.$$

### 2.3. Multipolar Representation with Mulliken Charges and Dipoles

The Mulliken decomposition of the electron density is:

(9) $$\rho_{A}\left(r\right)=\sum_{\mu ,\nu \in A}P^{\mu \nu }\chi_{\mu }\left(r\right)\chi_{\nu }\left(r\right)+\sum_{\mu \in A,\nu \notin A}P^{\mu \nu }\chi_{\mu }\left(r\right)\chi_{\nu }\left(r\right),$$

where $\rho_{A}\left(r\right)$ is the electron density assigned to atom *A*. In this expression, $\chi_{\mu }$ and $\chi_{\nu }$ refer to atomic basis functions, and $P^{\mu \nu }$ represent elements of the one-particle density matrix. From these atomic densities, we can calculate Mulliken charges and dipoles and use them to represent the QM subsystem,

(10) $$q_{A}^{\mathrm{Mul}}=Z_{A}-\int \rho_{A}\left(r\right)dr,$$

(11) $$\begin{array}{c} \mu_{A}^{\mathrm{Mul}}=\int \left(r-r_{A}\right)\rho_{A}\left(r\right)dr. \end{array}$$

When the multipolar expansion in Equation (5) is truncated at the monopolar level, the energy becomes:

(12) $$E_{\mathrm{QM}/\mathrm{MM}}^{\mathrm{II},\;\mathrm{MulC}}=\sum_{A}q_{A}^{\mathrm{Mul}}\phi_{A},$$

which leads to the MulC model, and the QM subsystem is represented by Mulliken charges only. On the other hand, if the multipole expansion is truncated at the dipolar level, Equation (5) becomes:

(13) $$E_{\mathrm{QM}/\mathrm{MM}}^{\mathrm{II},\;\mathrm{MulCD}}=\sum_{A}\left[q_{A}^{\mathrm{Mul}}\phi_{A}+\mu_{Ax}^{\mathrm{Mul}}E_{Ax}+\mu_{Ay}^{\mathrm{Mul}}E_{Ay}+\mu_{Az}^{\mathrm{Mul}}E_{Az}\right],$$

which leads to the MulCD model, and the QM subsystem is represented by Mulliken charges and dipoles.

### 2.4. Multipolar Representation with ESP Charges and Dipoles

Alternatively, we can represent the QM electron density with ESP charges and dipoles [35]. In general, ESP charges and dipoles, as denoted by $M_{Am}^{\mathrm{ESP}}$, are fitted to reproduce electrostatic potential $\phi_{s}$ on each point *s* of a pre-defined grid outside the QM subsystem,

(14) $$\sum_{A}\sum_{m}K_{Am,s}M_{Am}^{\mathrm{ESP}}=\phi_{s},$$

where the interaction tensor $K_{Am,s}$ has essentially the same definition as $T_{Am,B}$, except that $K_{Am,s}$ couples the QM atomic multipole moments to charges on the grid points *s* (instead of MM point charges). We can perform singular-value decomposition (SVD) to invert the interaction tensor and obtain the ESP multipoles according to:

(15) $$M_{Am}^{\mathrm{ESP}}=\sum_{s}{\left(K^{-1}\right)}_{Am,s}\phi_{s}.$$

Inserting these atomic multipoles into Equation (5), we get the following QM/MM electrostatic energy:

(16) $$E_{\mathrm{QM}/\mathrm{MM}}^{\mathrm{II},\;\mathrm{ESP}}=\sum_{A}\sum_{m}M_{Am}^{\mathrm{ESP}}F_{Am}\;$$

which can be similarly truncated at the charges or dipoles level to yield ESPC and ESPCD models for ab initio QM/MM electrostatics. Furthermore, the energy expansion can be rewritten as:

(17) $$\begin{array}{ccc} E_{\mathrm{QM}/\mathrm{MM}}^{\mathrm{II},\;\mathrm{ESP}} & = & \sum_{A}\sum_{m}\left[\sum_{s}{\left(K^{-1}\right)}_{Am,s}\phi_{s}\right]\left[\sum_{B}q_{B}T_{Am,B}\right]\\ & = & \sum_{s}\left[\sum_{A}\sum_{m}\sum_{B}q_{B}T_{Am,B}{\left(K^{-1}\right)}_{Am,s}\right]\phi_{s}\\ & = & \sum_{s}q_{s}\phi_{s}\;, \end{array}$$

where $q_{s}$ is

(18) $$q_{s}=\sum_{A}\sum_{m}\sum_{B}q_{B}T_{Am,B}{\left(K^{-1}\right)}_{Am,s}$$

Putting Equations (17) and (18) together, it simply means that all MM charges ($q_{B}$) are now projected onto the ESP grid and get represented by the projected charges ($q_{s}$) on this grid. This closely resembles the aforementioned SMBP method from Benighaus and Thiel [27,28], where remote MM charges are also approximated as a set of surface charges (on a sphere much further away from QM atoms).

### 2.5. A Hybrid Scheme with Switching Functions

In our hybrid scheme, density and multipole embedding schemes are used in the short- and long-range QM/MM electrostatic interactions, respectively. The energy is thus written as:

(19) $$E_{\mathrm{QM}/\mathrm{MM}}^{\mathrm{elec},\;\mathrm{hybrid}}=E_{\mathrm{QM}/\mathrm{MM}}^{\mathrm{elec},\;I}\left[q_{B}^{⩽}\right]+E_{\mathrm{QM}/\mathrm{MM}}^{\mathrm{elec},\;\mathrm{II}}\left[q_{B}^{>}\right]$$

where $q_{B}^{⩽}$ refers to MM charges within a cutoff distance $r_{\mathrm{off}}$ and $q_{B}^{>}$ represents those charges beyond the cutoff. Here, $r_{B}$, the distance between an MM point charge $q_{B}$ and its closest QM atom, would be used to determine to which region the charge belongs.

During geometry optimizations or molecular dynamics simulations, some MM atoms might cross the cutoff boundary and cause discontinuities in the QM/MM electrostatics energy in Equation (19). To smooth the transition, a switching function S(r) can be applied to divide each inner MM charge into two portions:

(20) $$\begin{array}{ccc} q_{B}^{⩽} & = & q_{B}^{I,⩽}+q_{B}^{II,⩽} \end{array}$$

(21) $$\begin{array}{ccc} q_{B}^{I,⩽} & = & S\left(r_{B}\right)q_{B}^{⩽} \end{array}$$

(22) $$\begin{array}{ccc} q_{B}^{II,⩽} & = & \left(1-S(r_{B})\right)q_{B}^{⩽} \end{array}$$

which interact with the QM density and multipoles, respectively,

(23) $$E_{\mathrm{QM}/\mathrm{MM}}^{\mathrm{elec},\;\mathrm{hybrid}-\mathrm{sw}}=E_{\mathrm{QM}/\mathrm{MM}}^{\mathrm{elec},\;I}\left[q_{B}^{I,⩽}\right]+E_{\mathrm{QM}/\mathrm{MM}}^{\mathrm{elec},\;\mathrm{II}}\left[q_{B}^{II,⩽}\;\;\cup \;\;q_{B}^{>}\right]$$

When no smoothing is applied, the “scaling” function is a Step function:

(24) $$S^{\mathrm{Step}}\left(r\right)=\left\{\begin{array}{cc} 1, & r⩽r_{\mathrm{off}},\\ 0, & r>r_{\mathrm{off}}. \end{array}\right.$$

In this study, we tested two smoothing functions widely used in MM simulations [36], including the Shift function:

(25) $$S^{\mathrm{Shift}}=\left\{\begin{array}{cc} {\left(1-{\left(r/r_{\mathrm{off}}\right)}^{2}\right)}^{2}, & r⩽r_{\mathrm{off}},\\ 0, & r>r_{\mathrm{off}}, \end{array}\right.$$

and the Switch function,

(26) $$S^{\mathrm{Switch}}\left(r\right)=\left\{\begin{array}{cc} 1, & r⩽r_{\mathrm{on}},\\ \frac{{\left(r_{\mathrm{off}}^{2}-r^{2}\right)}^{2}\left(r_{\mathrm{off}}^{2}+2r^{2}-3r_{\mathrm{on}}^{2}\right)}{{\left(r_{\mathrm{off}}^{2}-r_{\mathrm{on}}^{2}\right)}^{3}}\;, & r_{\mathrm{on}}<r⩽r_{\mathrm{off}},\\ 0, & r>r_{\mathrm{off}}, \end{array}\right.$$

with $r_{\mathrm{on}}$ set to be $0.75\;r_{\mathrm{off}}$ in this work. The long-range electrostatic correction (LREC) function [25,26] mentioned above:

(27) $$S^{\mathrm{LREC}}\left(r\right)=\left\{\begin{array}{cc} 1-{\left(2{\left(1-r/r_{\mathrm{off}}\right)}^{3}-3{\left(1-r/r_{\mathrm{off}}\right)}^{2}+1\right)}^{2}, & r⩽r_{\mathrm{off}},\\ 0, & r>r_{\mathrm{off}}. \end{array}\right.$$

was also considered.

## 3. Computational Details

**Figure 1:**
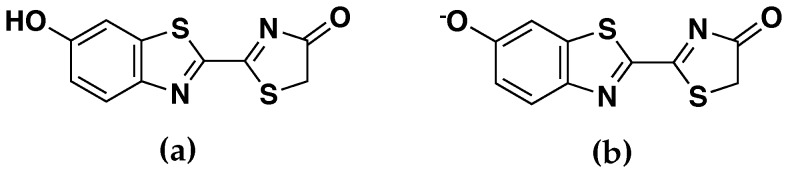
Structures of the (**a**) neutral (OLU) and (**b**) anionic (${\mathrm{OLU}}^{-}$) forms of oxyluciferin.

We benchmarked these hybrid embedding schemes on four test systems, namely the oxyluciferin molecule in its neutral (OLU) and anionic (${\mathrm{OLU}}^{-}$) forms (Figure 1) in both aqueous and enzyme (luciferase) environments. To collect a variety of structures for the benchmarking QM/MM calculations, classical MD simulations were first performed on the test systems. For the aqueous systems, a single OLU or ${\mathrm{OLU}}^{-}$ molecule was solvated in a cubic box of TIP3P water [37] with an initial size of 120 Å × 120 Å × 120 Å. For the enzyme systems, the initial structures were built on the X-ray crystal structure of Japanese firefly luciferase complexed with the substrate oxyluciferin and the cofactor AMP (PDB ID: 2D1R) [38] and solvated in a same-sized TIP3P water box. A counter ion was randomly added near the surface of the water box to neutralize the system when necessary. The enzyme and cofactor were modeled using the CHARMM36 force field [39,40], whereas the force field parameters for OLU and ${\mathrm{OLU}}^{-}$ were obtained using the CHARMM general force field (CGenFF) [41].

Classical MD simulations were conducted under periodic boundary conditions using NAMD 2.12 [42]. The particle mesh Ewald method [5] was employed to treat the electrostatic interactions, while the van der Waals interactions were truncated at a cutoff of 12 Å with a switch function applied starting from 10 Å. To be able to use a time step of 2 fs for the MD integration, the SHAKE algorithm [43] was used to constrain all bonds involving hydrogen atoms. After a series of minimizations and equilibrations, a 1-ns NVT Langevin dynamics was performed at 300 K with an equilibrated box size of 117 Å × 117 Å × 117 Å for each test system, during which the heavy atoms of OLU or ${\mathrm{OLU}}^{-}$ were restrained to the starting structure by a weak harmonic potential (1 kcal/mol/Å^2^). Cartesian coordinates were saved every 10 ps along the trajectory, which resulted in 100 structures for each QM/MM benchmarking calculation.

The standard electrostatic embedding scheme, where all MM charges interact directly with the QM electron density (as shown in Equation (2)), was used in the reference calculations. Since the objective of this study is to benchmark the embedding schemes in condensed-phase calculations, we chose to use a large supercell consisting of the center cell and all the image cells within three cell lengths away from the center cell, in order to mimic a periodic system. The OLU or ${\mathrm{OLU}}^{-}$ molecule in the center cell was defined as the QM subsystem, while the rest of the center cell and all the image cells were included in the MM subsystem. B3LYP/6-31+G* was used as the QM method, while the MM atoms, including the OLU or ${\mathrm{OLU}}^{-}$ molecules in the image cells, were represented by their partial charges from the C36/CGenFF/TIP3P force fields.

In QM/MM calculations using other embedding schemes, the same QM method was employed, while an atom-centered spherical cutoff around the QM subsystem was used to divide the MM subsystem into near- and far-field regions. Atom-based cutoffs were applied in cases where a switching function (Shift, Switch or LREC) was used, while group-based cutoffs were employed when a Step function was used to avoid artificial net charges in the near-field region. All the QM/MM calculations were done using a locally-modified version of Q-Chem 4.4 [44].

We benchmarked the electrostatic and polarization energies from different embedding schemes against the reference calculations. Specifically, we started from converged molecular orbitals (and thus electron density) from reference calculations. The QM/MM electrostatic energy was calculated according to Equation (23). The QM/MM polarization energy was computed as the energy difference between the first and last SCF cycles, which measures the distortion of molecular orbitals away from reference ones. This is clearly important, because capturing the polarization of the QM wave function caused by the MM environment is the entire reason for carrying out electrostatic embedding. Population analysis and time-dependent density functional theory (TDDFT) excited state calculations [45,46], which directly depend on the polarized wave function, were also performed to test how well different embedding schemes could reproduce the reference values.

## 4. Results and Discussion

### 4.1. Electrostatic and Polarization Energies

**Figure 2:**
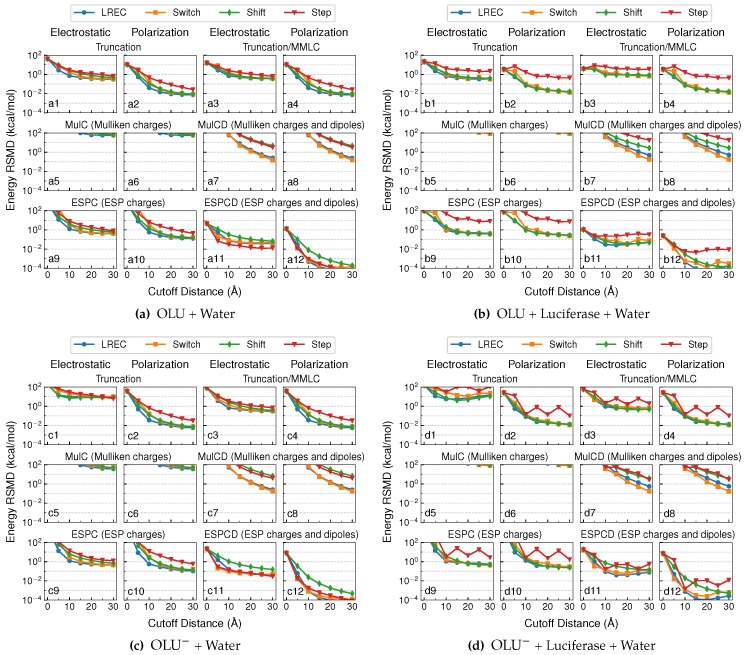
RMSD of electrostatic and polarization energies for neutral oxyluciferin in the (**a**) aqueous and (**b**) enzyme environments and anionic oxyluciferin in the (**c**) aqueous and (**d**) enzyme environments. All MM charges beyond a cutoff distance were removed from the QM/MM electrostatics calculation in the “Truncation” model, and the long-range electrostatics interaction between these far-field charges and the QM subsystem were described at the MM level in the “Truncation/MMLC” model. Near-field MM charges remained unchanged in the “Step” calculations, but scaled with the long-range electrostatic corrections (“LREC”), “Switch” and “Shift” calculations to ensure a continuous potential energy surface.

Figure 2 shows the root-mean-square deviations (RMSD) in the QM/MM electrostatic and polarization energies from the theoretical reference values for the neutral and anionic oxyluciferin systems in the aqueous and enzyme environments. The deviations, as averaged over 100 different configurations for each system, are shown at different cutoff distances, $r_{\mathrm{off}}$, ranging from 0–30 Å. Our goal was to identify embedding schemes that could produce both energy values within 0.1 kcal/mol from the reference values.

#### 4.1.1. Truncation and Truncation/MMLC Models

At a cutoff distance of 0 Å, both electrostatics and polarization energies were completely ignored within the “truncation” model. As such, the zero-cutoff values in Plots a1, b1, c1 and d1 in Figure 2 correspond to the average QM/MM electrostatic energy, which is the Coulomb interaction between the QM electron density (and nuclei) and all MM charges. The average QM/MM electrostatic energy fell in between 10 and 100 kcal/mol (34 kcal/mol in water and 21 kcal/mol in luciferase) for the neutral oxyluciferin molecule. Not surprisingly, it became much larger (179 kcal/mol in water and 151 kcal/mol in luciferase) for the oxyluciferin anion. The average QM/MM polarization energy, which is the energy for rotating the reference QM molecular orbitals back to gas-phase ones, also increased from 11 kcal/mol (Plot a2) to 38 kcal/mol (Plot c2) in aqueous solution and from 3 kcal/mol (Plot b2) to 26 kcal/mol (Plot d2) in the enzyme. This was also expected because, with a negative charge, the anion is usually more polarizable.

As the cutoff distance increased, the truncation model involved more and more near-field MM charges interacting directly with the QM electron density, while all far-field MM charges were removed. In other words, the long-range term in Equation (23) was neglected. Overall, the truncation model performed increasingly better for the neutral QM system (using the Step or switching functions) with larger distance cutoffs, and the electrostatic energy RMSD converged around 1 kcal/mol (Plots a1 and b1). Surprisingly, the calculations with the switching functions (and thus scaled near-field MM charges) outperformed the one with the Step function for the enzyme system (Plot b1). Similar results were observed in the QM/MM-LREC paper [25], where the QM subsystem of the test system was also neutral and the near-field MM charges were scaled down using the LREC switching function. This suggested that the scaling of near-field MM charges partially compensated for the neglecting of the far-field charges.

For the anionic QM system (Figure 2b,d), the truncation model performed much less well, where the electrostatic energy RMSD converged around 10 kcal/mol (Plots c1 and d1). This is understandable since the electrostatic potential of a charged QM region decays much more slowly than a neutral QM region (with only a net dipole).

Compared to the electrostatic energy, the truncation models reproduced the polarization energy much better. As shown in Plots a2, b2, c2 and d2 in Figure 2, when a switching function was used, the deviation in the polarization energy approached ∼0.1 kcal/mol already at a cutoff distance of 10 Å and further decayed to around 10^−2^ kcal/mol. Here, for the computation of polarization energy, QM/MM embedding schemes using switching functions performed several times better than using the Step function, again attesting to the usefulness of scaling down near-field MM charges to compensate for neglecting far-field MM charges.

Results in Figures S1 and S2 can help us understand why better results were obtained using the switching functions within the truncation model. As shown in Figure S1, positive atom-site potentials were found on all atoms in the ${\mathrm{OLU}}^{-}$ anion, reflecting a stabilization effect from all water molecules or protein residues. However, these atom-site potentials were noticeably underestimated when all MM atoms beyond a short cutoff distance were removed (see the top panels in Figure S1). To solvate an anion, water molecules tend to orient in a way that their hydrogen atoms are on average slightly closer to the anion than the oxygen atoms. When a switching function like LREC was applied, on average, the positive charge on hydrogen atoms received a larger scaling factor (i.e., closer to 1.0) than the negative charge on the oxygen atom of the same water molecule. As a result, the LREC-scaled MM charges added up to around 1.0 (see Figure S2), and this net positive charge helped bring the atom-site potential from near-field MM atoms closer to their reference values (see lower panels in Figure S1). In the case of the neutral OLU molecule, on the other hand, the atom-site fields (instead of potentials) had large errors upon the truncation, and these errors were then reduced by using a smoothing function.

In Figure 2, we also show the results from “Truncation/MMLC” calculations. Here, the same QM/MM calculations as the truncation model were performed first, meaning that the QM/MM polarization energies stayed the same (Plot a1, for example, is identical to 1b). Then, the long-range electrostatics between QM subsystem and far-field MM charges were accounted for at the MM level, using CGenFF charges on QM atoms. Within this scheme, the QM/MM electrostatics energies received no obvious improvement for the neutral QM systems (Plots a4 and b4). However, it was substantially improved in QM/MM calculations with the anionic QM system, where the deviation was reduced from ∼10 kcal/mol (Plots c1 and d1) to ∼1 kcal/mol (Plots c3 and d3).

#### 4.1.2. MulC and MulCD Models

For the MulC embedding, the electrostatic energy RMSD went beyond or slightly below 10^2^ kcal/mol (as shown in Plots a5, a6, b5, b6, c5, c6, d5 and d6 in Figure 2), even when large cutoffs were used for both the anionic and neutral QM systems. When the dipoles were included to interact with far-field MM charges, as in the MulCD embedding scheme, the results were much improved. However, in order to achieve a target accuracy of 0.1 kcal/mol, a large cutoff (>30 Å) and LREC/Switch functions were needed (see Plots a7, a8, b7, b8, c7, c8, d7 and d8 in Figure 2). This again confirms the inappropriateness of using Mulliken charges to account for long-range ai-QM/MM electrostatics, as discovered by Holden and Herbert [16,17].

#### 4.1.3. ESPC and ESPCD Models

For ESPC embedding, it performed poorly with short cutoffs. At larger cutoffs (>10 Å), its performance in terms of QM/MM electrostatic energy was found to be similar to that of the truncation model for the case with a neutral QM region (Plot a9 vs. a1 in Figure 2). For cases with an anionic QM subsystem, ESPC produced smaller (but still significant) errors (ca. 1 kcal/mol) in the QM/MM electrostatic energy (Plots c9 and d9) than the truncation model (ca. 10 kcal/mol, Plots c1 and d1 in Figure 2).

Much better performance was observed from the ESPCD embedding scheme, which outperformed all other embedding schemes at cutoff distances larger than 10 Å. At this cutoff distance, the computed QM/MM electrostatics energies using the LREC/Switch functions deviated from the reference values by no more than 0.1 kcal/mol (see a11, b11, c11 and d11 in Figure 2), thus achieving our target accuracy. An even higher accuracy of ∼10^−3^ kcal/mol can be seen from Plots a12, b12, c12 and d12 in Figure 2 for computed QM/MM polarization energies using the LREC/Switch functions and a cutoff distance beyond 10 Å, which meant the QM wavefunction deviated little from the reference ones.

For the ESPCD model, the Shift function led to slower convergence (than LREC/Switch) for both QM/MM electrostatics and polarization energies. A Step function, on the other hand, delivered a convergence behavior that was similar to LREC/Switch functions in aqueous solution, where it actually led to slightly more accurate results for the solvated neutral oxyluciferin molecule (Plot a11 in Figure 2). However, the Step function converged rather poorly in the enzyme environment, where a large variation in the net charge in the near-field MM region occurred with the cutoff distances (see Figure S2). Therefore, for a more consistent performance, LREC/Switch functions were more preferable.

### 4.2. Population Analysis and Excitation Energies

To test the influence of the electrostatics embedding schemes on the calculated properties of the QM subsystems, we also performed population analysis for the anionic oxyluciferin systems and computed its TDDFT excitation energies in aqueous and luciferase environments. A suitable QM/MM embedding scheme was expected to reproduce atomic charges within 0.01 $e^{-}$ and vertical excitation energies within 10^−2^ eV from the reference values.

**Figure 3:**
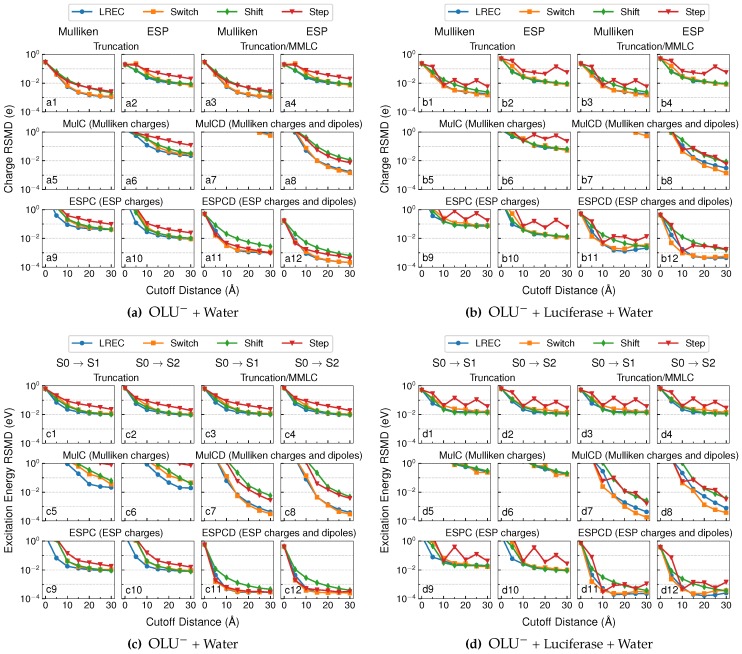
RMSD of Mulliken and ESP charges for the anionic oxyluciferin systems in the (**a**) aqueous and (**b**) enzyme environments; RMSD of excitation energies for the anionic oxyluciferin systems in the (**c**) aqueous and (**d**) enzyme environments.

The population analysis and excitation state calculations results are summarized in Figure 3. Since the truncation/MMLC model only added classical long-range electrostatics energies to the truncation model, it did not affect the electronic structure of the QM subsystem. Therefore, the plots for atomic populations and excitation energies in Figure 3 were identical between the two models.

As expected, the deviations of the atomic charges coincided with the corresponding polarization energies. For the truncation model, LREC/Switch switching functions are shown in Plots a1, a2, b1, and b2 in Figure 3 to yield a several-fold reduction in the deviation in the Mulliken/ESP charges than a simple truncation using the Step function. The MulC, MulCD and ESPC embedding models produced much less satisfactory results, at least in terms of the Mulliken atomic charges. Consistent with our observations above, the ESPCD model yielded the best performance, with both Mulliken and ESP charges deviating by less than 0.01 $e^{-}$ on average from reference values beyond a cutoff distance of 10 Å (see Plots a11, a12, b11 and b12 in Figure 3). This verified that the ECPCD model well reproduced the reference QM wavefunctions.

For excited state calculations, when combined with LREC/Switch functions, the truncation model could approach a 10^−2^ eV accuracy at a 15 Å cutoff (see Plots c1, c2, d1 and d1 in Figure 3). The MulC model performed poorly for excited state calculations, especially for the enzyme system where the RMSDs of excitation energies were found to be always above 0.1 eV at all cutoff distances (see Plots d5 and d6 in Figure 3). The ESPC model displayed a similar performance to the truncation model. Surprisingly, the MulCD model delivered rather accurate excitation energies (RMSD < 10^−2^ eV) beyond a 15 Å cutoff. The ESPCD model with LREC/Switch/Shift functions demonstrated a superior performance: the corresponding vertical excitation energies fell within 10^−2^ eV from the reference values already at a 5 Å cutoff, and the deviation further reduced to 10^−3^ eV beyond a 10 Å cutoff.

### 4.3. Computational Timings

Within our hybrid scheme, the timing of QM/MM electrostatics evaluation would be completely dominated by the short-range portion. The timing contribution from long-range QM/MM electrostatics is marginal, because it requires only the computation of the total electrostatic potential/field from the far-field MM charges at QM atom sites, which is relatively less computationally intensive even without a reciprocal-space acceleration. The cost is also unaffected by the use of a switching function, because, at a given cutoff distance, the switching scheme changes only the value (rather than the number) of near-field MM charges.

For short-range electrostatics, three-center one-electron Coulomb integrals (or their derivatives) between basis function pairs and each near-field MM charge are needed in an energy (force) evaluation. Therefore, the cost is expected to scale linearly with the number of near-field MM charges, which in turn scales roughly cubically with the cutoff distance.

This was confirmed by the timings in Table 1 for one of our test systems: oxyluciferin anion in aqueous environment. The timings for short-range electrostatics energy and force evaluations increased rapidly with the cutoff distance. Beyond 30 Å, a combined QM/MM electrostatics energy/force evaluation would take more than 50 s, which became comparable to the cost of gas-phase SCF energy (∼300 s) and gradient (∼60 s) evaluations. Therefore, since our ESPCD scheme allows the use of a 10 Å cutoff distance, the cost of QM/MM electrostatic evaluations will be kept at a negligible cost compared to the SCF energy and force evaluations.

## 5. Conclusions

In this work, we benchmarked several embedding schemes for long-range QM/MM electrostatic interactions. Here are our main observations from our ai-QM/MM calculations on neutral and anionic oxyluciferin in aqueous and enzyme environments:

- The long-range QM/MM electrostatic interactions can be rather significant. At cutoff distances of 30 Å, their average contribution was found to be still around 10 kcal/mol with the anionic QM subsystem and around 1 kcal/mol with the neutral QM subsystem.
- In truncated QM/MM calculations, where only MM charges within a cutoff distance are kept, like Fang et al. [25], we found it to be beneficial to use LREC/Switch functions to scale down MM charge values to compensate for neglecting all far-field MM charges.
- The MulC model should generally be avoided because it can cause extremely large errors in QM/MM electrostatic and polarization energies. While the MulCD model offered an improvement upon the MulC model, its use is still not recommended because large distance cutoffs are needed and because the Mulliken charges on QM atoms obtained with this model still looked erroneous.
- While the ESPC model performed significantly better than MulC, it polarized the QM region only as well as the truncation model (as indicated by atomic charge populations, and vertical excitation energies) when LREC or Switch functions were used. Its only significant advantage over the truncation model occurred with the computation of QM/MM electrostatics energy with an anionic QM subsystem (the oxyluciferin anion).
- The ESPCD model yielded the best performances. At a 10 Å cutoff distance, it reproduces QM/MM electrostatics energy within 0.1 kcal/mol, polarization energy within 10^−3^ kcal/mol and TDDT vertical excitation energy within 10^−3^ eV from the reference values. Therefore, ESPCD with LREC/Switch smoothing functions and a 10 Å cutoff would be our recommended combination for a hybrid representation for the QM subsystem in the treatment of short-range and long-range QM/MM electrostatics.
- Besides avoiding discontinuity at the cutoff distance, a LREC/Switch smoothing function between the near- and far-field interactions can also lead to better results in most cases, and thus should be applied in general.

On the other hand, this work has several limitations:

- Only the oxyluciferin systems were studied using very short MD simulations. Testing on longer simulations and more systems, including enzymatic reactions, needs to be carried out for more general conclusions.
- Our ESPCD scheme should be readily extendable to ai-QM/MM PBC calculations, where both ESP charges and dipoles are used to represent the QM subsystem in the long-range electrostatics calculations. However, this needs to be thoroughly tested.
- We used the standard ESP grid (discretized four layers of vdW surfaces) in the computation of ESP charges and dipoles. Other grids should be explored.
- Only energy values were reported. The corresponding analytical gradient has yet to be coded.
- The treatment of short-range QM/MM electrostatics in this work should be combined with models for avoiding over-polarization by accounting for the QM/MM charge penetration effect [47,48].
- The treatment of long-range QM/MM electrostatics should be extended to the use of advanced population models for the QM electron density, such as D-RESP [9], 3D-fitted ESP [49], and CM5 models [50].
- For the MM region, the TIP3P water model and C36 protein force field were employed. Therefore, it would be interesting to see if other force fields might offer a physically better description for the QM/MM electrostatics/polarization interactions.
- Our focus was placed on QM/MM electrostatics/polarization energies. Therefore, we have not considered QM/MM vdW and charge-transfer interactions, which can also significantly affect the simulation results.
- We studied how to more quickly converge QM/MM electrostatic energy with the number of MM charges included in the short-range evaluation. Therefore, we have not addressed the equally important issue of how to achieve quicker convergence of QM/MM results with the size of the QM region.

We are working on addressing some of these limitations.

## Figures and Tables

**Table 1:** Number of near-field MM charges and CPU time (in seconds) for QM/MM electrostatic energy and force evaluations at different cutoff distances. The QM region is the oxyluciferin anion, which is described by the B3LYP/6-31+G* level of theory. The MM region is a 117 Å × 117 Å × 117 Å unit cell of TIP3P water molecules. Obtained using a single Intel Xeon E5-2650 core at 2.3 GHz.

Cutoff (Å)	# (Charges)	Time (s)		Cutoff (Å)	# (Charges)	Time (s)	
		Energy	Force			Energy	Force
5	144	0.1	0.4	25	9297	3.7	28.2
10	837	0.3	2.0	30	15,291	5.8	42.5
15	2418	0.9	5.8	60	107,535	39.4	262.7
20	5121	2.2	15.3	Unit Cell	166,114	58.2	403.6

## Supplementary Materials

The following are available online. Figure S1: Atom-site potential from near-field MM charges versus atom-site potential from all MM charges, Figure S2: Sum of near-field MM charges at different cutoff distances.
