# Supplementary material for: Representation of the QM Subsystem for Long-Range Electrostatic Interaction in Non-Periodic Ab Initio QM/MM Calculations
Source: Molecules. 2018 Sep 29;23(10):2500. doi: 10.3390/molecules23102500 (PMC6222767; doi:10.3390/molecules23102500)
Supplement: Supplementary file 1 [file molecules-23-02500-s001.pdf]

Supplementary Information for "Representation of the QM Subsystem for Long-Range Electrostatic Interaction in Non-Periodic Ab Initio QM/MM Calculations"

Section S1. ANIONIC QM SUBSYSTEM: OLU$^-$

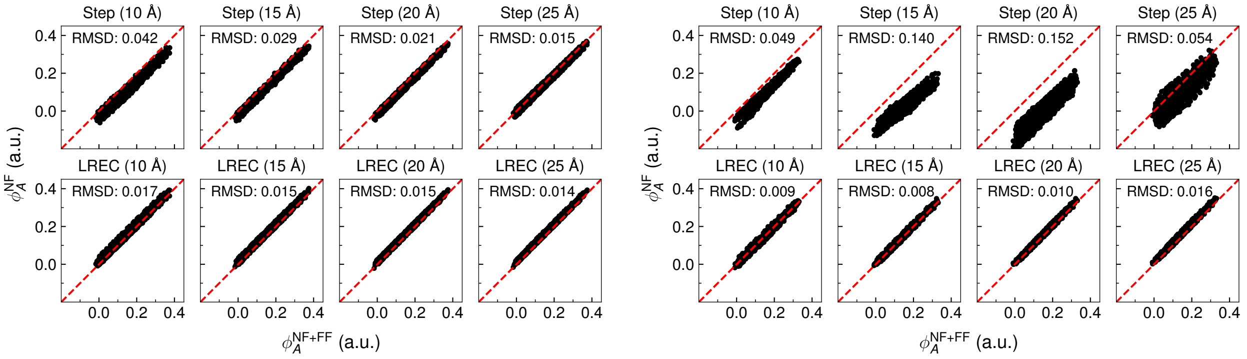

(a) OLU$^-$ + Water **(b)** OLU$^-$ + Luciferase + Water

Figure S1. Atom-site potential from near-field MM charges *versus* atom-site potential from all MM charges. Each panel correspond to a different cutoff distance and either Step (near-field MM charges are not scaled) or LREC (near-field MM charges are scaled) scheme.

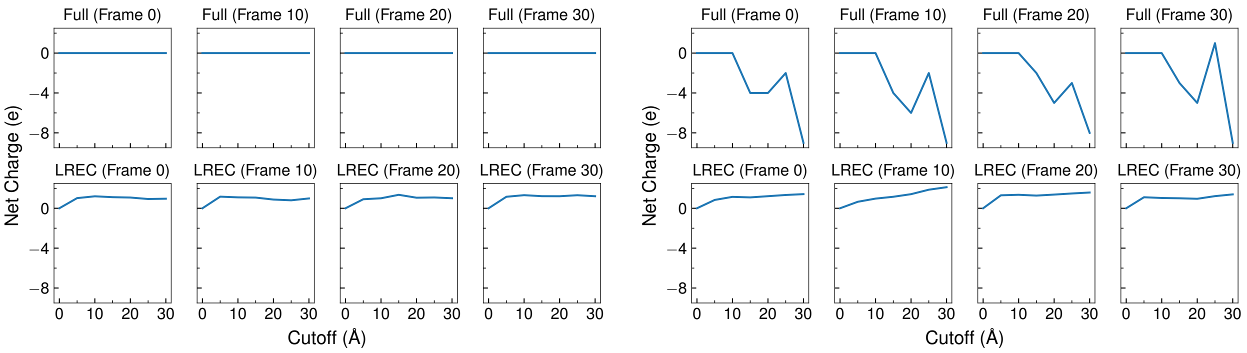

(a) OLU$^-$ + Water **(b)** OLU$^-$ + Luciferase + Water

Figure S2. Sum of near-field MM charges at different cutoff distances. Each panel refers to either Step (near-field MM charges are not scaled) or LREC (near-field MM charges are scaled) scheme.
